# *cis*-Tetra­aqua­bis­{5-[4-(1*H*-imidazol-1-yl-κ*N* ^3^)phen­yl]tetra­zolido}manganese(II) dihydrate

**DOI:** 10.1107/S1600536812010380

**Published:** 2012-03-14

**Authors:** Xin Wang, Shi-Wei Yan, Suo-Cheng Chang, Yan-Chen Liang, Fu-Tian Zhang

**Affiliations:** a College of Chemistry and Chemical Engineering, Southwest University, Chongqing 400715, People’s Republic of China

## Abstract

In the title compound, [Mn(C_10_H_7_N_6_)_2_(H_2_O)_4_]·2H_2_O, the complex unit comprises an Mn^2+^ ion, coordinated by two imidazole N atoms from *cis*-related monodentate 5-[4-(imidazol-1-yl)phen­yl]tetra­zolide ligands and four water mol­ecules, together with two water mol­ecules of solvation. The Mn^2+^ ion lies on a twofold rotation axis and has a slightly distorted octa­hedral geometry. The mol­ecules are connected by O—H⋯N and O—H⋯O hydrogen bonds involving both coordinated and solvent water mol­ecules, generating a three-dimensional structure. Two C atoms of the imidazole ring of the ligand are each disordered over two sites with occupancy factors of 0.75 and 0.25.

## Related literature

For general background to the use of nitro­gen-containing ligands in the construction of supra­molecular coordination compounds, see: Qi *et al.* (2008 ▶). For the structure of the anhydrous *trans*-isomer of the title complex, see: Cheng (2011 ▶).
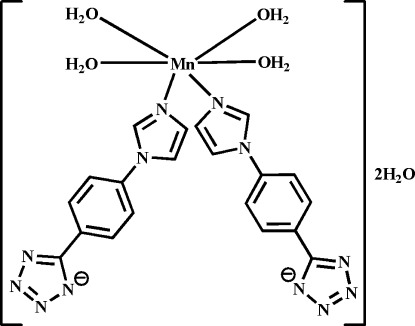


## Experimental

### 

#### Crystal data

[Mn(C_10_H_7_N_6_)_2_(H_2_O)_4_]·2H_2_O
*M* *_r_* = 585.47
Monoclinic, 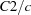

*a* = 19.239 (3) Å
*b* = 13.141 (2) Å
*c* = 13.417 (2) Å
β = 129.912 (2)°
*V* = 2601.8 (7) Å^3^
*Z* = 4
Mo *K*α radiation
μ = 0.57 mm^−1^
*T* = 296 K
0.50 × 0.45 × 0.35 mm

#### Data collection

Bruker APEX CCD area-detector diffractometer
Absorption correction: multi-scan (*SADABS*; Sheldrick, 1996 ▶) *T*_min_ = 0.764, *T*_max_ = 0.826
7759 measured reflections
2962 independent reflections
2246 reflections with *I* > 2σ(*I*)
*R*_int_ = 0.027

#### Refinement

*R*[*F* ^2^ > 2σ(*F* ^2^)] = 0.037
*wR*(*F* ^2^) = 0.099
*S* = 1.04
2962 reflections
219 parameters
H atoms treated by a mixture of independent and constrained refinement
Δρ_max_ = 0.21 e Å^−3^
Δρ_min_ = −0.27 e Å^−3^

### 

Data collection: *SMART* (Bruker, 2001 ▶); cell refinement: *SAINT* (Bruker, 2001 ▶); data reduction: *SAINT*; program(s) used to solve structure: *SHELXS97* (Sheldrick, 2008 ▶); program(s) used to refine structure: *SHELXL97* (Sheldrick, 2008 ▶); molecular graphics: *SHELXTL* (Sheldrick, 2008 ▶); software used to prepare material for publication: *SHELXL97*.

## Supplementary Material

Crystal structure: contains datablock(s) I, global. DOI: 10.1107/S1600536812010380/zs2184sup1.cif

Structure factors: contains datablock(s) I. DOI: 10.1107/S1600536812010380/zs2184Isup2.hkl

Additional supplementary materials:  crystallographic information; 3D view; checkCIF report

## Figures and Tables

**Table 1:** Hydrogen-bond geometry (Å, °)

*D*—H⋯*A*	*D*—H	H⋯*A*	*D*⋯*A*	*D*—H⋯*A*
O1*W*—H11*W*⋯O3*W*	0.79 (4)	1.91 (4)	2.690 (4)	169 (2)
O1*W*—H12*W*⋯O3*W*^i^	0.82 (3)	1.95 (3)	2.762 (3)	168 (4)
O2*W*—H21*W*⋯N4^ii^	0.79 (4)	2.07 (4)	2.847 (4)	169 (3)
O2*W*—H22*W*⋯N5^iii^	0.85 (3)	1.97 (3)	2.812 (3)	170 (4)
O3*W*—H31*W*⋯N3^iv^	0.83 (4)	1.97 (3)	2.786 (3)	167 (3)
O3*W*—H31*W*⋯N4^iv^	0.83 (4)	2.62 (3)	3.308 (3)	142 (3)
O3*W*—H32*W*⋯N6^v^	0.82 (3)	1.95 (3)	2.757 (3)	172 (2)

Symmetry codes: (i) 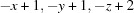; (ii) 
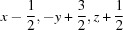
; (iii) 
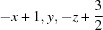
; (iv) 
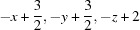
; (v) 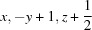.

**Fig. 1.**
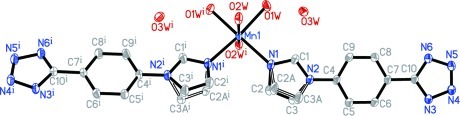
Molecular configuration and atom numbering scheme for the title complex, showing the partial disorder in the imidaziole ring of the ligand. Displacement ellipsoids are drawn at the 30% probability level. For symmetry code (i): -x - 1, y, -z + 5/2.

## supplementary crystallographic information

### Comment

In recent years, much study has been focused on using nitrogen-containing ligands and 1,4-benzenedicarboxylic acid to construct supramolecular coordination compounds. The reason is that these supramolecular coordination assemblies exhibit not only a variety of architectures but also have potential applications as functional materials (Qi *et al.*, 2008). In this paper, we report the synthesis and structure of the title complex [Mn(timb)_2_(H_2_O)_4_] . 2H_2_O, {Htimb = 5-[4-imidazol-1-yl)phenyl]tetrazole}, synthesized by the hydrothermal reaction of manganese(II) acetate with Htimb in the presence of 1,4-benzenedicarboxylic acid.

In the title compound the coordination polyhedron comprises a Mn^2+^ ion, two monodentate *cis*-related timb^-^ ligands and four coordinated water molecules, together with two water molecules of solvation (Fig. 1). The Mn^2+^ lies on a twofold rotation axis and the complex has a slightly distorted octahedral geometry. Both the coordinated and solvent water molecules form intermolecular O—H···O and O—H···N hydrogen-bonding interactions (Table 1) to form a three-dimensional supramolecular network. Two atoms of the imidazole ring (C2, C3) are disordered over two sites (C2*A* and C3*A*, respectively), with occupancy factors of 0.75 and 0.25. The structure of the anhydrous *trans*-isomer of the title complex has previously been reported (Cheng, 2011).

### Experimental

A mixture of Mn(OAc)_2_ (0.098 g, 0.4 mmol), 5-[4-imidazol-1-yl)phenyl]tetrazole (Htimb) (0.064 g, 0.3 mmol), 1,4-benzendicarboxylic acid (0.066 g, 0.4 mmol) and water (9 ml) was stirred for 30 min in air. The mixture was then transferred to a 18 ml Teflon-lined hydrothermal bomb. The bomb was kept at 433 K for 75 h under autogenous pressure. The product was washed with distilled water and dried, giving the yellow title compound.

### Refinement

Hydrogen atoms bound to C atoms were placed in calculated positions and treated as riding on their parent atoms, with C—H = 0.93 Å and with *U*_iso_(H) = 1.2*U*_eq_(C). Water H atoms were located in a difference-Fourier analysis and both positional and isotropic displacement parameters were allowed to refine. Atom pairs C2, C2*A* and C3, C3*A* represent disordered atoms of the imidazole ring of the ligand, having site occupancies of 0.75 and 0.25.

### Crystal data

**Table:**

[Mn(C_10_H_7_N_6_)_2_(H_2_O)_4_]·2H_2_O	*F*(000) = 1212
*M**_r_* = 585.47	*D*_x_ = 1.495 Mg m^−^^3^
Monoclinic, *C*2/*c*	Mo *K*α radiation, λ = 0.71073 Å
Hall symbol: -C 2yc	Cell parameters from 7759 reflections
*a* = 19.239 (3) Å	θ = 2.1–27.5°
*b* = 13.141 (2) Å	µ = 0.57 mm^−^^1^
*c* = 13.417 (2) Å	*T* = 296 K
β = 129.912 (2)°	Block, yellow
*V* = 2601.8 (7) Å^3^	0.50 × 0.45 × 0.35 mm
*Z* = 4	

### Data collection

**Table:**

Bruker APEX CCD area-detector diffractometer	2962 independent reflections
Radiation source: fine-focus sealed tube	2246 reflections with *I* > 2σ(*I*)
Graphite monochromator	*R*_int_ = 0.027
φ and ω scans	θ_max_ = 27.5°, θ_min_ = 2.1°
Absorption correction: multi-scan (*SADABS*; Sheldrick, 1996)	*h* = −24→24
*T*_min_ = 0.764, *T*_max_ = 0.826	*k* = −17→16
7759 measured reflections	*l* = −12→17

### Refinement

**Table:**

Refinement on *F*^2^	Primary atom site location: structure-invariant direct methods
Least-squares matrix: full	Secondary atom site location: difference Fourier map
*R*[*F*^2^ > 2σ(*F*^2^)] = 0.037	Hydrogen site location: inferred from neighbouring sites
*wR*(*F*^2^) = 0.099	H atoms treated by a mixture of independent and constrained refinement
*S* = 1.04	*w* = 1/[σ^2^(*F*_o_^2^) + (0.0453*P*)^2^ + 1.08*P*] where *P* = (*F*_o_^2^ + 2*F*_c_^2^)/3
2962 reflections	(Δ/σ)_max_ = 0.002
219 parameters	Δρ_max_ = 0.21 e Å^−^^3^
0 restraints	Δρ_min_ = −0.27 e Å^−^^3^

### Special details

**Table:**

Geometry. Bond distances, angles etc. have been calculated using the rounded fractional coordinates. All su's are estimated from the variances of the (full) variance-covariance matrix. The cell esds are taken into account in the estimation of distances, angles and torsion angles
Refinement. Refinement of *F*^2^ against ALL reflections. The weighted *R*-factor *wR* and goodness of fit *S* are based on *F*^2^, conventional *R*-factors *R* are based on *F*, with *F* set to zero for negative *F*^2^. The threshold expression of *F*^2^ > σ(*F*^2^) is used only for calculating *R*-factors(gt) *etc*. and is not relevant to the choice of reflections for refinement. *R*-factors based on *F*^2^ are statistically about twice as large as those based on *F*, and *R*- factors based on ALL data will be even larger.

### Fractional atomic coordinates and isotropic or equivalent isotropic displacement parameters (Å^2^)

**Table:**

	*x*	*y*	*z*	*U*_iso_*/*U*_eq_	Occ. (<1)
Mn1	0.50000	0.64609 (3)	1.25000	0.0381 (2)	
O1W	0.50199 (14)	0.52591 (12)	1.13954 (19)	0.0494 (6)	
O2W	0.35299 (11)	0.62992 (13)	1.09094 (17)	0.0487 (6)	
N1	0.53170 (13)	0.76342 (13)	1.16074 (18)	0.0471 (6)	
N2	0.58359 (12)	0.81830 (13)	1.06351 (18)	0.0439 (6)	
N3	0.72694 (13)	0.88544 (13)	0.73642 (18)	0.0457 (6)	
N4	0.73655 (13)	0.84944 (13)	0.65176 (19)	0.0476 (6)	
N5	0.70484 (12)	0.75736 (14)	0.61811 (17)	0.0476 (7)	
N6	0.67343 (12)	0.72992 (13)	0.67890 (17)	0.0452 (6)	
C1	0.54836 (16)	0.74146 (16)	1.0828 (2)	0.0499 (8)	
C2	0.5739 (3)	0.8554 (3)	1.2134 (4)	0.0586 (16)	0.750
C3	0.6063 (3)	0.8903 (3)	1.1558 (4)	0.0600 (15)	0.750
C4	0.61025 (13)	0.81709 (14)	0.98504 (19)	0.0371 (6)	
C5	0.64673 (17)	0.90308 (16)	0.9751 (2)	0.0500 (8)	
C6	0.67239 (17)	0.90067 (16)	0.8996 (2)	0.0503 (8)	
C7	0.66198 (13)	0.81344 (14)	0.83318 (19)	0.0358 (6)	
C8	0.62672 (15)	0.72795 (16)	0.8463 (2)	0.0469 (7)	
C9	0.60114 (15)	0.72957 (16)	0.9219 (2)	0.0495 (8)	
C10	0.68777 (13)	0.80981 (14)	0.75070 (19)	0.0358 (6)	
C2A	0.5081 (10)	0.8671 (8)	1.1261 (14)	0.067 (5)	0.250
C3A	0.5379 (10)	0.9036 (8)	1.0642 (14)	0.070 (5)	0.250
O3W	0.63171 (12)	0.47411 (14)	1.12748 (17)	0.0481 (6)	
H1	0.53660	0.67790	1.04430	0.0600*	
H5	0.65400	0.96230	1.01900	0.0600*	
H2	0.57970	0.88850	1.27980	0.0710*	0.750
H3	0.63730	0.95100	1.17400	0.0710*	0.750
H9	0.57770	0.67120	0.93000	0.0590*	
H11W	0.5443 (19)	0.510 (2)	1.146 (2)	0.065 (9)*	
H12W	0.457 (2)	0.524 (2)	1.062 (3)	0.069 (9)*	
H21W	0.3226 (18)	0.6277 (18)	1.111 (3)	0.063 (8)*	
H22W	0.329 (2)	0.666 (2)	1.023 (3)	0.078 (10)*	
H6	0.69710	0.95870	0.89330	0.0600*	
H8	0.62010	0.66830	0.80350	0.0560*	
H21A	0.47760	0.90570	1.14540	0.0810*	0.250
H31A	0.52910	0.96810	1.02920	0.0830*	0.250
H31W	0.6792 (19)	0.508 (2)	1.171 (3)	0.070 (9)*	
H32W	0.6465 (17)	0.415 (2)	1.150 (2)	0.060 (8)*	

### Atomic displacement parameters (Å^2^)

**Table:**

	*U*^11^	*U*^22^	*U*^33^	*U*^12^	*U*^13^	*U*^23^
Mn1	0.0532 (3)	0.0388 (2)	0.0477 (3)	0.0000	0.0440 (2)	0.0000
O1W	0.0568 (11)	0.0558 (10)	0.0539 (11)	0.0025 (8)	0.0439 (10)	−0.0081 (8)
O2W	0.0539 (10)	0.0631 (10)	0.0514 (10)	0.0093 (8)	0.0440 (9)	0.0084 (8)
N1	0.0636 (12)	0.0482 (10)	0.0584 (11)	−0.0029 (9)	0.0524 (10)	0.0005 (8)
N2	0.0619 (11)	0.0410 (9)	0.0575 (11)	−0.0042 (8)	0.0515 (10)	−0.0015 (8)
N3	0.0657 (12)	0.0424 (9)	0.0591 (12)	−0.0070 (8)	0.0539 (11)	−0.0051 (8)
N4	0.0638 (12)	0.0487 (10)	0.0596 (11)	−0.0038 (9)	0.0530 (11)	−0.0039 (9)
N5	0.0626 (12)	0.0505 (11)	0.0531 (11)	−0.0060 (9)	0.0479 (10)	−0.0068 (8)
N6	0.0611 (11)	0.0448 (10)	0.0509 (10)	−0.0094 (8)	0.0456 (10)	−0.0075 (8)
C1	0.0740 (16)	0.0430 (11)	0.0625 (14)	−0.0077 (11)	0.0574 (14)	−0.0020 (10)
C2	0.089 (3)	0.052 (2)	0.080 (3)	−0.017 (2)	0.075 (2)	−0.0164 (19)
C3	0.092 (3)	0.0465 (18)	0.084 (3)	−0.0215 (19)	0.076 (3)	−0.0183 (18)
C4	0.0430 (11)	0.0414 (10)	0.0436 (11)	0.0005 (8)	0.0354 (10)	0.0034 (8)
C5	0.0795 (16)	0.0366 (11)	0.0686 (15)	−0.0057 (10)	0.0634 (14)	−0.0049 (10)
C6	0.0797 (16)	0.0372 (11)	0.0717 (15)	−0.0079 (10)	0.0658 (15)	−0.0016 (10)
C7	0.0413 (11)	0.0400 (10)	0.0390 (11)	−0.0004 (8)	0.0317 (10)	0.0022 (8)
C8	0.0650 (14)	0.0426 (11)	0.0553 (13)	−0.0131 (10)	0.0488 (13)	−0.0109 (10)
C9	0.0695 (15)	0.0439 (12)	0.0624 (14)	−0.0187 (11)	0.0548 (13)	−0.0082 (10)
C10	0.0420 (11)	0.0375 (10)	0.0399 (11)	0.0011 (8)	0.0318 (10)	0.0019 (8)
C2A	0.108 (10)	0.058 (6)	0.100 (9)	0.028 (7)	0.096 (9)	0.021 (6)
C3A	0.120 (11)	0.045 (5)	0.106 (10)	0.021 (6)	0.101 (9)	0.020 (6)
O3W	0.0543 (11)	0.0420 (9)	0.0597 (10)	0.0019 (8)	0.0420 (9)	−0.0048 (8)

### Geometric parameters (Å, º)

**Table:**

Mn1—O1W	2.183 (2)	N4—N5	1.299 (3)
Mn1—O2W	2.203 (2)	N5—N6	1.339 (4)
Mn1—N1	2.263 (2)	N6—C10	1.329 (3)
Mn1—O1W^i^	2.183 (2)	C2—C3	1.347 (9)
Mn1—O2W^i^	2.203 (2)	C2A—C3A	1.36 (3)
Mn1—N1^i^	2.263 (2)	C4—C5	1.381 (4)
O1W—H12W	0.82 (3)	C4—C9	1.371 (3)
O1W—H11W	0.79 (4)	C5—C6	1.383 (5)
O2W—H21W	0.79 (4)	C6—C7	1.385 (3)
O2W—H22W	0.85 (3)	C7—C10	1.476 (4)
O3W—H32W	0.82 (3)	C7—C8	1.381 (3)
O3W—H31W	0.83 (4)	C8—C9	1.383 (4)
N1—C2	1.372 (5)	C1—H1	0.9300
N1—C1	1.308 (4)	C2—H2	0.9300
N1—C2A	1.417 (11)	C2A—H21A	0.9300
N2—C4	1.437 (4)	C3—H3	0.9300
N2—C3	1.388 (5)	C3A—H31A	0.9300
N2—C3A	1.428 (16)	C5—H5	0.9300
N2—C1	1.331 (4)	C6—H6	0.9300
N3—C10	1.333 (3)	C8—H8	0.9300
N3—N4	1.348 (3)	C9—H9	0.9300
			
O1W—Mn1—O2W	80.99 (9)	N1—C1—N2	113.9 (2)
O1W—Mn1—N1	90.32 (8)	N1—C2—C3	110.1 (4)
O1W—Mn1—O1W^i^	87.31 (8)	N1—C2A—C3A	111.5 (15)
O1W—Mn1—O2W^i^	90.98 (8)	N2—C3—C2	106.5 (4)
O1W—Mn1—N1^i^	168.63 (10)	N2—C3A—C2A	103.2 (9)
O2W—Mn1—N1	99.64 (8)	N2—C4—C9	119.9 (2)
O1W^i^—Mn1—O2W	90.98 (8)	C5—C4—C9	119.9 (3)
O2W—Mn1—O2W^i^	168.93 (7)	N2—C4—C5	120.27 (19)
O2W—Mn1—N1^i^	87.94 (8)	C4—C5—C6	119.6 (2)
O1W^i^—Mn1—N1	168.63 (10)	C5—C6—C7	121.2 (2)
O2W^i^—Mn1—N1	87.94 (8)	C6—C7—C10	122.0 (2)
N1—Mn1—N1^i^	94.10 (8)	C8—C7—C10	119.89 (19)
O1W^i^—Mn1—O2W^i^	80.99 (9)	C6—C7—C8	118.1 (3)
O1W^i^—Mn1—N1^i^	90.32 (8)	C7—C8—C9	121.1 (2)
O2W^i^—Mn1—N1^i^	99.64 (8)	C4—C9—C8	120.1 (2)
H11W—O1W—H12W	108 (3)	N3—C10—C7	125.22 (18)
Mn1—O1W—H11W	125.7 (18)	N6—C10—C7	123.8 (2)
Mn1—O1W—H12W	115 (2)	N3—C10—N6	111.0 (2)
H21W—O2W—H22W	112 (4)	N2—C1—H1	123.00
Mn1—O2W—H21W	117 (2)	N1—C1—H1	123.00
Mn1—O2W—H22W	117 (3)	N1—C2—H2	125.00
H31W—O3W—H32W	107 (3)	C3—C2—H2	125.00
Mn1—N1—C1	124.09 (14)	C3A—C2A—H21A	124.00
Mn1—N1—C2A	133.7 (9)	N1—C2A—H21A	124.00
C1—N1—C2	103.8 (3)	C2—C3—H3	127.00
C1—N1—C2A	98.1 (8)	N2—C3—H3	127.00
Mn1—N1—C2	126.8 (2)	N2—C3A—H31A	129.00
C3A—N2—C4	121.6 (7)	C2A—C3A—H31A	128.00
C1—N2—C3A	101.7 (8)	C4—C5—H5	120.00
C3—N2—C4	127.3 (3)	C6—C5—H5	120.00
C1—N2—C4	126.86 (19)	C5—C6—H6	119.00
C1—N2—C3	104.6 (3)	C7—C6—H6	119.00
N4—N3—C10	104.79 (18)	C9—C8—H8	120.00
N3—N4—N5	109.4 (2)	C7—C8—H8	119.00
N4—N5—N6	109.5 (2)	C8—C9—H9	120.00
N5—N6—C10	105.31 (19)	C4—C9—H9	120.00
			
O1W—Mn1—N1—C1	−4.3 (2)	N4—N3—C10—N6	0.1 (3)
O1W—Mn1—N1—C2	−154.2 (4)	N4—N3—C10—C7	−179.0 (2)
O2W—Mn1—N1—C1	−85.2 (2)	N3—N4—N5—N6	0.1 (3)
O2W—Mn1—N1—C2	124.9 (4)	N4—N5—N6—C10	0.0 (3)
O2W^i^—Mn1—N1—C1	86.7 (2)	N5—N6—C10—N3	−0.1 (3)
O2W^i^—Mn1—N1—C2	−63.3 (4)	N5—N6—C10—C7	179.1 (2)
N1^i^—Mn1—N1—C1	−173.8 (2)	N1—C2—C3—N2	0.2 (5)
N1^i^—Mn1—N1—C2	36.3 (4)	N2—C4—C5—C6	179.8 (2)
Mn1—N1—C1—N2	−166.66 (17)	C9—C4—C5—C6	1.1 (4)
C2—N1—C1—N2	−11.0 (3)	N2—C4—C9—C8	−180.0 (2)
Mn1—N1—C2—C3	161.0 (3)	C5—C4—C9—C8	−1.3 (4)
C1—N1—C2—C3	6.3 (5)	C4—C5—C6—C7	0.2 (4)
C3—N2—C1—N1	11.3 (4)	C5—C6—C7—C8	−1.2 (4)
C4—N2—C1—N1	179.0 (2)	C5—C6—C7—C10	179.1 (2)
C1—N2—C3—C2	−6.5 (5)	C6—C7—C8—C9	1.0 (4)
C4—N2—C3—C2	−174.1 (3)	C10—C7—C8—C9	−179.3 (2)
C1—N2—C4—C5	−179.5 (2)	C6—C7—C10—N3	3.6 (4)
C1—N2—C4—C9	−0.8 (4)	C6—C7—C10—N6	−175.4 (2)
C3—N2—C4—C5	−14.5 (4)	C8—C7—C10—N3	−176.1 (2)
C3—N2—C4—C9	164.2 (3)	C8—C7—C10—N6	4.9 (4)
C10—N3—N4—N5	−0.1 (3)	C7—C8—C9—C4	0.2 (4)

Symmetry code: (i) −*x*+1, *y*, −*z*+5/2.

### Hydrogen-bond geometry (Å, º)

**Table:**

*D*—H···*A*	*D*—H	H···*A*	*D*···*A*	*D*—H···*A*
O1*W*—H11*W*···O3*W*	0.79 (4)	1.91 (4)	2.690 (4)	169 (2)
O1*W*—H12*W*···O3*W*^ii^	0.82 (3)	1.95 (3)	2.762 (3)	168 (4)
O2*W*—H21*W*···N4^iii^	0.79 (4)	2.07 (4)	2.847 (4)	169 (3)
O2*W*—H22*W*···N5^iv^	0.85 (3)	1.97 (3)	2.812 (3)	170 (4)
O3*W*—H31*W*···N3^v^	0.83 (4)	1.97 (3)	2.786 (3)	167 (3)
O3*W*—H31*W*···N4^v^	0.83 (4)	2.62 (3)	3.308 (3)	142 (3)
O3*W*—H32*W*···N6^vi^	0.82 (3)	1.95 (3)	2.757 (3)	172 (2)

Symmetry codes: (ii) −*x*+1, −*y*+1, −*z*+2; (iii) *x*−1/2, −*y*+3/2, *z*+1/2; (iv) −*x*+1, *y*, −*z*+3/2; (v) −*x*+3/2, −*y*+3/2, −*z*+2; (vi) *x*, −*y*+1, *z*+1/2.
